# Targeting SMPDL3B to Ameliorate Radiation- and Cisplatin-Induced Renal Toxicity

**DOI:** 10.3390/cells15020205

**Published:** 2026-01-22

**Authors:** Anis Ahmad, Shamroop Kumar Mallela, Saba Ansari, Mohammed Alnukhali, Sandra Merscher, Alla Mitrofanova, Youssef H. Zeidan, Alan Pollack, Alessia Fornoni, Brian Marples

**Affiliations:** 1 Department of Radiation Oncology, University of Miami Miller School of Medicine, Sylvester Comprehensive Cancer Center, Miami, FL 33136, USA; 2 Department of Biochemistry and Molecular Biology, University of Miami Miller School of Medicine, Miami, FL 33136, USA; 3 Katz Family Division of Nephrology and Hypertension, Department of Medicine, and Peggy and Harold Katz Family Drug Discovery Center, University of Miami Miller School of Medicine, Miami, FL 33136, USA; 4 Department of Radiation Oncology Baptist Health, Lynn Cancer Institute, Boca Raton, FL 33437, USA; 5 Department of Radiation Oncology, University of Rochester Medical Center, 601 Elmwood Ave. Box 647, Rochester, NY 14642, USA

**Keywords:** SMPDL3B, ceramide-1-phosphate, radiation nephropathy, cisplatin, podocyte injury, glomerular basement membrane, lipid metabolism, nephrotoxicity

## Abstract

**Highlights:**

**What are the main findings?**

- Combined radiation + cisplatin reduces podocyte SMPDL3B, driving podocyte loss, GBM thickening, mesangial expansion, fibrosis, albuminuria, and accumulation of long-chain C1P linked to inflammation/cell death.
- Podocyte-specific SMPDL3B overexpression protects kidney structure and function after genotoxic injury and normalizes abnormal C1P accumulation.

**What are the implications of the main findings?**

- SMPDL3B is a key regulator of podocyte survival and sphingolipid balance during cancer therapy-associated kidney stress, making it a promising target to prevent nephrotoxicity.
- Enhancing SMPDL3B activity/expression may expand the therapeutic window for radiotherapy and platinum chemotherapy, improving tumor control while preserving long-term kidney health.

**Abstract:**

Kidney toxicity remains a major dose-limiting complication of radiation therapy and platinum-based chemotherapy, yet the molecular determinants of renal susceptibility and resilience to these genotoxic treatments are incompletely understood. Podocytes are particularly vulnerable to such insults, and emerging evidence implicates lipid dysregulation in podocyte injury. This study investigated the role of sphingomyelin phosphodiesterase acid-like 3B (SMPDL3B), a podocyte-enriched lipid-modulating enzyme, in radiation- and cisplatin-induced nephrotoxicity. Using a doxycycline-inducible, podocyte-specific SMPDL3B transgenic mouse model, renal injury was assessed following focal kidney irradiation, cisplatin administration, or their combination through functional assays, histopathology, ultrastructural analysis, immunofluorescence, and targeted lipidomics. Combined radiation and cisplatin exposure markedly reduced podocyte SMPDL3B expression, accompanied by podocyte depletion, glomerular basement membrane remodeling, proteinuria, and impaired renal function. These structural and functional abnormalities were associated with the selective accumulation of long-chain ceramide-1-phosphate species. In contrast, podocyte-specific induction of SMPDL3B preserved glomerular architecture, maintained renal function, and prevented pathological ceramide-1-phosphate elevation. Collectively, these findings identify SMPDL3B as a key regulator of podocyte stability and lipid homeostasis during chemoradiation stress. Enhancing SMPDL3B activity may represent a mechanistically grounded strategy to mitigate treatment-induced kidney injury while preserving anticancer efficacy.

## 1. Introduction

Radiation therapy (RT) and cisplatin-based chemotherapy are essential components in the curative management of pelvic, abdominal, thoracic, and head-and-neck malignancies, with concurrent administration often employed to increase locoregional tumor control and extend patient survival [1,2]. However, nephrotoxicity emerges as a predominant dose-limiting factor for both modalities, necessitating regimen modifications that may undermine antitumor efficacy [3,4]. Radiation nephropathy unfolds gradually, progressing from subtle tubular and vascular insults to overt glomerulosclerosis and interstitial fibrosis, with clinical signs such as hypertension, anemia, and azotemia typically manifesting 6–24 months after exposure [5,6].

Cisplatin remains a cornerstone of therapy for many solid tumors, yet its clinical utility is significantly limited by the kidney’s heightened vulnerability to injury [7]. Across multiple clinical cohorts, 20–33% of patients develop acute kidney injury (AKI) following treatment [8,9]. This acute toxicity stems from cisplatin’s rapid accumulation within renal epithelial cells and the resultant mitochondrial damage, oxidative stress, and DNA injury that drive cell death and inflammation [10,11]. Although partial recovery may occur, it is frequently incomplete. Longitudinal analyses reveal that approximately 25–40% of patients experience sustained declines in glomerular filtration, and 10–20% advance to chronic kidney disease (CKD), with nearly 11.8% progressing to end-stage kidney disease (ESKD) or death in long-term follow-up [12]. Susceptibility is further exacerbated by comorbid conditions and cumulative cisplatin exposure [12]. While comprehensive long-term pharmacokinetic datasets remain limited [13], available evidence indicates that cisplatin and its metabolites may persist in renal tissue, perpetuating tubular stress and nephron loss long after therapy concludes [13,14]. Taken together, these findings highlight that cisplatin nephrotoxicity is not confined to the acute treatment window but represents a chronic and clinically meaningful threat to kidney health, underscoring the urgent need for mechanistically driven renoprotective approaches that preserve both renal function and anticancer efficacy [15,16].

Podocytes, the specialized visceral epithelial cells integral to the glomerular filtration barrier (GFB), uphold selective permeability through their elaborate foot processes and slit diaphragms, preventing macromolecular leakage [17,18]. Compromised podocyte architecture, evidenced by effacement, cytoskeletal disruption, apoptosis, or detachment, precipitates proteinuria and propels glomerulosclerotic progression, a hallmark not only of idiopathic glomerulopathies like focal segmental glomerulosclerosis (FSGS) and diabetic kidney disease (DKD) but also of radiation- and cisplatin-mediated nephropathies [5,19,20,21]. Contemporary investigations have identified sphingolipid (SL) dysmetabolism as a cardinal modulator of podocyte adaptation to genotoxic and inflammatory stressors [22,23]. Dysregulated SL species, including ceramides, sphingomyelins, and sphingosine-1-phosphate (S1P), orchestrate apoptotic, fibrotic, and vasoactive cascades; aberrant ceramide accumulation and S1P pathway perturbations exacerbate podocyte attrition in DKD and FSGS, fostering inflammation and filtration barrier compromise [22]. Notably, ceramide-1-phosphate (C1P), a phosphorylated ceramide derivative, has surfaced as a prognostic indicator of renal deterioration, with its upregulation inciting arachidonic acid liberation, prostaglandin biosynthesis, and NF-κB-driven inflammatory amplification in preclinical AKI and CKD models [24,25].

Sphingomyelin phosphodiesterase acid-like 3B (SMPDL3B), a glycosylphosphatidylinositol-anchored ectoenzyme expressed on podocyte membranes among other cell types, hydrolyzes sphingomyelin to generate ceramide and phosphocholine, thereby refining lipid raft composition and transducing signals for cytoskeletal homeostasis, insulin responsiveness, and innate immunity [26]. Experimental abrogation of SMPDL3B heightens susceptibility to oxidative and DNA-damaging insults, aggravating podocyte effacement and genotoxic responses in radiation-challenged models through unchecked p53 activation and caspase cascades [27,28]. Conversely, SMPDL3B deficiency uncouples proteinuria from tubulointerstitial advancement in hereditary glomerulopathies, affirming its pivotal governance of barrier function [19]. Employing a doxycycline-inducible, podocyte-restricted SMPDL3B transgenic (SMP TG) murine framework, this investigation elucidates SMPDL3B’s mechanistic footprint on sphingolipid landscape reconfiguration, glomerular ultrastructural safeguarding, and renal functional sustenance under RT-cisplatin synergy. Augmenting foundational observations of SMPDL3B’s anti-genotoxic protection [27,28] and sphingolipid equilibrium oversight [22], we hypothesize that podocyte-specific augmentation of SMPDL3B limits ceramide-1-phosphate (C1P)-associated inflammatory signaling, thereby reducing podocyte injury and attenuating progression from AKI to CKD. If confirmed, these findings would support SMPDL3B and its lipid effectors as candidate biomarkers and therapeutic targets to predict and mitigate nephrotoxicity, including potential approaches such as rituximab repurposing or development of SMPDL3B-enhancing agents, in patients receiving cisplatin-sensitized radiotherapy [4,11,23].

## 2. Materials and Methods

### 2.1. Materials and Reagents

For cell culture needs, RPMI 1640 medium (catalog number 10-040-CV) was obtained from Corning to support human podocyte growth, while DMEM/F-12 (1:1) GlutaMAX medium (catalog number 11320-033), heat-inactivated fetal bovine serum (FBS; catalog number 26140-079), and 100× penicillin–streptomycin solution (catalog number 15140-163) were sourced from Gibco–Thermo Fisher Scientific (Waltham, MA, USA) for murine podocyte maintenance. The insulin–transferrin–selenium (ITS) supplement (catalog number 25-800-CR) came from Corning to aid in cell differentiation and survival. Reagents for conditionally immortalized podocyte differentiation included cyclic di-adenosine monophosphate sodium salt (catalog number SML1231) and 9-oxo-10(9H)-acridineacetic acid (γ-secretase inhibitor XX; catalog number 17927), both purchased from Sigma-Aldrich (St. Louis, MO, USA), along with recombinant mouse interferon-γ (catalog number 315-05) from PeproTech (Cranbury, NJ, USA). In vivo experiments utilized doxycycline-hyclate-impregnated rodent chow at 200 mg/kg (catalog number TD.120037) from Envigo Teklad Diets (Madison, WI, USA), cisplatin (catalog number P4394) and streptozotocin (catalog number S0130) from Sigma-Aldrich, corn oil as a vehicle (catalog number C8267) from Sigma-Aldrich, and sodium citrate buffer pH 6.0 in 10× concentrate (catalog number S4641) also from Sigma-Aldrich. Molecular biology and protein quantification involved the RNeasy Mini Kit (catalog number 74106) from Qiagen (Hilden, Germany) for RNA extraction, GoTaq Green Master Mix (catalog number M7123) from Promega (Madison, WI, USA) for PCR amplification, qScript cDNA SuperMix (catalog number 95048-500) and PerfeCTa SYBR Green FastMix ROX (catalog number 95073-012) from QuantaBio (Beverly, MA, USA) for reverse transcription and quantitative PCR, the Pierce BCA Protein Assay Kit (catalog number 23225) from Thermo Fisher Scientific for protein measurement, precast 4–15% Criterion TGX SDS-PAGE gels (catalog number 5671084) and Trans-Blot Turbo Mini PVDF Transfer Packs (catalog number 1704156) from Bio-Rad (Hercules, CA, USA) for electrophoresis and blotting. Assessments of renal function relied on the Mouse Albumin ELISA Kit (catalog number E90-134) from Bethyl Laboratories (Montgomery, TX, USA) and the Creatinine Companion Assay Kit (catalog number 0420-500) from Stanbio Laboratory (Boerne, TX, USA). Histology and immunohistochemistry supplies included 70-µm and 100-µm cell strainers (catalog numbers 352350 and 352360) from Falcon–Corning (Corning, NY, USA), Hydrogen Peroxide Block and UltraVision Quanto Detection System (catalog number TL-060-QHL) from Thermo Fisher Scientific (Waltham, MA, USA), DAB Quanto Chromogen and Substrate (catalog number TA-060-QHDX) from Thermo Fisher Scientific, and the In Situ Cell Death Detection Kit, TMR red for TUNEL assay (catalog number 12156792910) from Roche (Basel, Switzerland). Primary antibodies comprised rabbit anti-SMPDL3B (custom or catalog number GWB-MM123A from GenWay Biotech (San Diego, CA, USA), guinea pig anti-synaptopodin (catalog number 163004 from Synaptic Systems) (Göttingen, Germany), rabbit anti-WT1 (catalog number ab89969 from Abcam, Cambridge, UK), and rabbit anti-desmin (catalog number 4024 from Cell Signaling, Danvers, MA, USA), with secondary antibodies being Alexa Fluor 488- and 568-conjugated donkey anti-rabbit/anti-guinea pig IgG at 1:500 dilution from Invitrogen–Thermo Fisher Scientific (Waltham, MA, USA). Additional items, such as Amicon Ultra-0.5 mL centrifugal filters with 3 kDa cutoff (catalog number UFC500396) from MilliporeSigma (Burlington, MA, USA), along with other routine laboratory chemicals and disposables of analytical purity, were procured from Thermo Fisher Scientific, Corning, or Sigma-Aldrich as required.

### 2.2. Methods

#### 2.2.1. Study Approval

All animal experiments described in this study were conducted in strict accordance with the ethical principles and regulatory requirements outlined by the National Institutes of Health Guide for the Care and Use of Laboratory Animals (8th edition), the Animal Research: Reporting of In Vivo Experiments (ARRIVE) 2.0 guidelines, and the Translational Nephrology Science for new Medications (TRANSFORM) consensus recommendations for preclinical kidney research. The complete study protocol, including mouse housing conditions, randomization procedures, treatment administration, and endpoint analyses, received formal approval from the Institutional Animal Care and Use Committee (IACUC #17-174, 20-164, 22-053, 23-052) of the University of Miami Miller School of Medicine. All procedures were performed by trained personnel under aseptic conditions, with continuous monitoring of animal welfare, and early euthanasia criteria were applied when predefined humane endpoints were reached.

#### 2.2.2. Animal Housing

Mice were housed in a specific pathogen-free barrier facility managed by the Division of Veterinary Resources at the University of Miami Miller School of Medicine. Animals were maintained in individually ventilated cages (Tecniplast Green Line Sealsafe Plus, West Chester, PA, USA) on a 12-h light/dark cycle (lights on 07:00–19:00), at a controlled ambient temperature of 22 ± 1 °C and relative humidity of 50–60%. Corn-cob bedding (Andersons Lab Bedding, Maumee, OH, USA) and nesting material were provided for enrichment. Reverse-osmosis-purified water and sterilized Teklad Global 18% Protein Rodent Diet (Envigo #2018S) (Indianapolis, IN, USA) were available ad libitum. Cages were changed twice weekly under laminar-flow conditions. Daily welfare assessments were performed by veterinary technicians, with additional weekly evaluations by the research team. Body weight was measured bi-weekly, and food/water consumption, general activity, posture, coat quality, and respiratory effort were recorded daily. Humane endpoints (≥20% body-weight loss, severe lethargy, hunched posture, or labored breathing) triggered immediate euthanasia. At study termination, mice were deeply anesthetized with ketamine (100 mg/kg) and xylazine (10 mg/kg) administered intraperitoneally, followed by transcardial perfusion with ice-cold 1× phosphate-buffered saline. Death was confirmed by cervical dislocation in accordance with the 2020 AVMA Guidelines for the Euthanasia of Animals. Post-perfusion, the kidney was collected and immersion-fixed for histologic and ultrastructural studies, or snap-frozen in liquid nitrogen for molecular analyses. Animals were randomized to experimental groups to ensure balanced distribution of sex, age, and litter across cohorts. All procedures were conducted under Institutional Animal Care and Use Committee.

#### 2.2.3. Generation of Doxycycline-Inducible, Podocyte-Specific Smpdl3b Overexpressing Mice

The podocyte-specific, doxycycline-inducible *Smpdl3b* transgenic mouse line (pSMPTg) used in this study was developed in collaboration with Dr. Alessia Fornoni’s laboratory at the University of Miami Miller School of Medicine, as previously described in detail [29]. In brief, full-length human *SMPDL3B* cDNA carrying an N-terminal Myc-DDK tag was amplified using high-fidelity PCR with gene-specific primers [29] (sequences listed in Supplementary Table S1 of the original report). The resulting amplicon was resolved by agarose gel electrophoresis; the band corresponding to the complete open reading frame was excised, blunt-ended, and purified using Qiagen Gel Extraction and PCR Cleanup kits. The processed cDNA fragment was then directionally cloned into the blunt-ended pTRE3G vector downstream of a Tet-responsive element (TRE3G). The final TetO-Myc-DDK-SMPDL3B cassette was released by AseI restriction digestion, purified, and microinjected into the pronuclei of fertilized C57BL/6J oocytes at the University of Miami Transgenic Core Facility. Founder animals harboring the transgene (SMPTg) were identified by genomic PCR and bred to the established podocin-rtTA driver line (stock #013156, The Jackson Laboratory, Bar Harbor, ME, USA) to achieve podocyte-restricted, doxycycline-dependent expression. Double-transgenic offspring (podocin-rtTA × SMPTg, hereafter designated pSMPTg) were backcrossed onto a pure C57BL/6J background for >8 generations. Overexpression of Myc-DDK-tagged SMPDL3B in podocytes was induced by administering custom doxycycline-containing chow (2000 ppm; Envigo Teklad Diets) starting at 7 weeks of age and continued throughout the experimental period. Induction efficiency and podocyte specificity were routinely confirmed by quantitative RT-PCR, Western blotting, and immunofluorescence co-staining for Myc/DDK, SMPDL3B, and podocyte markers (synaptopodin, WT1). All animal procedures involving this line were approved under the University of Miami IACUC protocol.

#### 2.2.4. Experimental Groups and Treatments

Male and female mice were randomly assigned to experimental groups. Two experimental cohorts were used. In the Cisplatin plus RT cohort, mice were assigned to: (1) NT/vehicle control, (2) RT (single dose of 4 Gy), (3) CP (3 mg/kg, i.p.), or (4) CP + RT (concurrently). In the SMPDL3B transgenic cohort, both WT (wild-type, non-transgenic) and TG (transgenic, SMPDL3B-overexpressing) were assigned to groups WT non-treated (WT-NT) and WT irradiated with a single dose of 14Gy (WT + RT), and were compared to TG non-treated (TG-NT) and TG irradiated with a single dose of 14Gy (TG + RT) to evaluate the protective effect of podocyte-specific SMPDL3B overexpression.

Focal renal irradiation was performed using a CT-image-guided small-animal arc radiation therapy platform (iSMAART, 225 kVp, 13 mA) [30]. For immobilization during irradiation, mice received ketamine (100 mg/kg) and ylazine (10 mg/kg) intraperitoneally. Depending on the experimental design, a single 4 Gy or 14 Gy bilateral renal dose was delivered using the same CT-guided iSMAART system and shielding configuration. Custom-designed lead shielding restricted the radiation field to both kidneys while sparing the rest of the body, and dose uniformity was confirmed using Gafchromic EBT3 film [5,31]. Cisplatin (Sigma-Aldrich, P4394) was administered once intraperitoneally. The selected irradiation and cisplatin doses were based on prior pilot experiments and published models demonstrating reproducible chronic glomerular and tubulointerstitial injury with acceptable early survival [4,27]. Animals were monitored weekly for body weight, general health, and welfare indicators. At 20 weeks post-treatment, a time point chosen to capture late radiation nephropathy and chronic cisplatin-induced injury [32], animals were deeply anesthetized with ketamine/xylazine and perfused transcardially with ice-cold PBS to remove intravascular blood. Euthanasia was completed via cervical dislocation in accordance with AVMA guidelines. Blood was collected by cardiac puncture, and kidneys were harvested immediately for histological evaluation, transmission electron microscopy, and lipidomic analyses. Sample sizes (*n* = 3–5 per group) were calculated using G*Power 3.1 to achieve 80% statistical power (α = 0.05) to detect ≥30% differences in podocyte number or albumin-to-creatinine ratio. All animal procedures were conducted under protocols approved by the University of Miami Institutional Animal Care and Use Committee and followed NIH guidelines for the care and use of laboratory animals.

#### 2.2.5. Estimation of Glomerular Filtration Rate (GFR)

Glomerular filtration rate was measured non-invasively in conscious mice immediately before euthanasia using a transdermal fluorescein isothiocyanate (FITC)-sinistrin clearance system (MediBeacon GmbH, Mannheim, Germany), as previously reported with minor modifications [33,34,35]. Twenty-four hours before measurement, the dorsal fur between the shoulder blades was removed using an electric clipper, followed by a depilatory cream (Nair) to ensure optimal skin contact. On the day of assessment, mice were briefly anesthetized with 1.5–2.5% isoflurane in oxygen, and a pre-calibrated transdermal fluorescence detector was affixed to the shaved area using a double-sided adhesive patch. After a 5-min stabilization period under continued light anesthesia, a single bolus of FITC-sinistrin (7.5 mg/100 g body weight, diluted in sterile 0.9% saline) was administered via tail-vein injection. Isoflurane was discontinued immediately after injection, and fluorescence decay was recorded transcutaneously for 90–120 min while mice recovered and moved freely in their home cage. Raw fluorescence data were transferred to a dedicated workstation, background-corrected, and analyzed using MediBeacon Studio software (version 4.2 or higher). GFR was calculated from the FITC-sinistrin elimination half-life (t_1_/_2_) using a validated single-compartment model and an empirically derived conversion factor specific to C57BL/6 mice: GFR (µL/min) = 15,384.6/t_1_/_2_ (min). Only recordings with R^2^ ≥ 0.95 for the mono-exponential fit were accepted for final analysis. This transdermal approach avoids repeated blood sampling and provides a highly correlated result with gold-standard plasma clearance methods [33,34,35].

#### 2.2.6. Urine Collection and Albumin-to-Creatinine Ratio (ACR) Measurement

Spot urine samples were collected from individually housed mice in the early morning (08:00–10:00), both at baseline and at predefined post-treatment time points (20 weeks after irradiation or cisplatin administration). Mice were gently placed over a clean plastic surface, and spontaneously voided urine was aspirated directly into low-protein-binding microcentrifuge tubes using a sterile pipette. Samples were immediately placed on ice, centrifuged at 2000× *g* for 5 min at 4 °C to remove debris, aliquoted, and stored at −80 °C until analysis. Urinary albumin concentration was quantified in duplicate using a mouse-specific sandwich ELISA kit (Bethyl Laboratories, cat# E90-134) according to the manufacturer’s protocol, with a standard curve ranging from 7.8 to 500 ng/mL. Urinary creatinine was measured in parallel using a modified Jaffé picrate-based colorimetric assay (Stanbio Laboratory, cat# 0420-500) adapted to a 96-well plate format; samples were diluted 1:20 in distilled water, and absorbance was read at 510 nm after 10 min incubation at 37 °C. Intra- and inter-assay coefficients of variation were <6% and <9%, respectively, for both assays. The albumin-to-creatinine ratio was calculated for each animal as ACR (µg/mg) = urinary albumin (µg/mL) ÷ urinary creatinine (mg/mL) and used as the primary index of glomerular barrier integrity.

#### 2.2.7. Blood Sample Analysis

Serum biochemical markers of renal function were quantified using validated, high-precision methodologies. Blood urea nitrogen (BUN) was measured in duplicate on a Roche Cobas c311 automated chemistry analyzer using the kinetic urease–glutamate dehydrogenase method at the Comparative Laboratory Core Facility, University of Miami Miller School of Medicine. Serum creatinine concentration was determined by isotope-dilution liquid chromatography–tandem mass spectrometry (LC-MS/MS) at the UAB-UCSD O’Brien Core Center for Acute Kidney Injury Research (University of Alabama at Birmingham, NIH P30 DK079337), following a previously established reference protocol that provides superior accuracy over conventional Jaffé or enzymatic assays [36]. Briefly, 20 µL of serum was spiked with C313-creatinine internal standard, proteins were precipitated with acetonitrile, and the supernatant was analyzed on an Agilent 1290 Infinity II UHPLC (Agilent Technologies, Santa Clara, CA, USA) coupled to a 6470 triple-quadrupole mass spectrometer in positive electrospray mode. Quantification was performed using the *m*/*z* 114 → 44 transition for creatinine and *m*/*z* 117 → 47 for the internal standard. Calibration curves were linear (r^2^ > 0.999) over 0.05–20 mg/dL. Results from both assays were reported in mg/dL and used to assess longitudinal changes in renal excretory function.

#### 2.2.8. Periodic Acid–Schiff (PAS) Staining and Mesangial Matrix Quantification

Four-micrometer paraffin-embedded kidney sections were deparaffinized in xylene (three changes, 5 min each) and rehydrated through a descending ethanol series (100, 95, 70%, 3 min each) followed by distilled water. Slides were oxidized in 0.5% periodic acid (Sigma-Aldrich #395132) for 10 min at room temperature, rinsed thoroughly in distilled water (three changes), and incubated with Schiff’s reagent (Sigma-Aldrich #3952016) for 15 min in the dark. After washing under running tap water for 5 min to develop the magenta color, sections were counterstained with Gill’s hematoxylin (30 s), blued in Scott’s tap water substitute (30 s), dehydrated through ascending ethanol, cleared in xylene, and permanently mounted with Entellan (Millipore). Bright-field images were acquired on an Olympus BX53 upright microscope equipped with a DP74 color camera using a 40× objective (numerical aperture 0.95). For each animal, a minimum of 60 superficial and juxtamedullary glomeruli were randomly selected and digitally captured under identical illumination and exposure settings. Mesangial matrix expansion was scored semi-quantitatively using a validated 0–4 scale (0 = no expansion; 1 = <25%; 2 = 25–50%; 3 = 50–75%; 4 = >75% of glomerular tuft area occupied by PAS-positive material) by two experienced renal pathologists who were blinded to experimental group assignment. Inter-observer agreement was >92%; discordant cases were resolved by consensus review. The average mesangial score per animal was used for statistical comparisons [37,38]. All image analysis and scoring were performed using Fiji/ImageJ (v2.14.0) with the color deconvolution plugin for objective PAS signal isolation when required [39].

#### 2.2.9. Picrosirius Red (PSR) Staining and Quantification of Interstitial Fibrosis

Paraffin-embedded kidney sections (3–5 μm) were first deparaffinized in xylene and subsequently rehydrated through a descending ethanol series. The rehydrated sections were immersed in Picrosirius Red solution (0.1% Sirius Red dissolved in saturated picric acid) for 60 min at room temperature to label collagen-rich extracellular matrix. Following staining, slides were gently rinsed twice with 0.5% acetic acid to remove unbound dye, dehydrated through graded ethanol, cleared in xylene, and coverslipped using a permanent mounting medium. Bright-field micrographs were obtained using an Olympus BX41 microscope (Tokyo, Japan) at 40× magnification, and additional high-resolution images were collected under polarized light to enhance the birefringence of collagen fibers. Fibrosis was quantified using ImageJ software by applying standardized color-thresholding parameters to isolate Picrosirius Red–positive regions. The extent of fibrosis was expressed as the proportion of collagen-stained area relative to the total tissue area examined [40].

#### 2.2.10. WT1 Immunostaining for Podocyte Quantification

Kidney sections (4 µm) were deparaffinized in xylene, rehydrated through a graded ethanol series, and subjected to heat-induced antigen retrieval in citrate buffer (pH 6.0) at 110 °C for 10 min. Tissue permeabilization was achieved using 0.3% Triton X-100 in PBS for 10 min at room temperature, followed by a blocking step with 5% BSA and 2.5% FBS in PBS for 1 h. Sections were then incubated overnight at 4 °C with the anti-WT1 primary antibody (1:300). After primary incubation, slides were washed and exposed to an Alexa fluorophore-conjugated secondary antibody (1:500) for 1 h at room temperature. Following final PBS washes, sections were dehydrated and mounted with antifade medium. Fluorescent images were captured using a Leica SP5 inverted confocal microscope equipped with a 40× water-immersion objective (Leica Microsystems, Mannheim, Germany). Quantification of WT1-positive podocyte nuclei was performed in individual glomeruli using ImageJ software by two independent, blinded observers [41].

#### 2.2.11. Synaptopodin and SMPDL3B Immunofluorescence Staining and Quantitative Fluorescence Analysis

Kidney sections (4 µm) were deparaffinized in xylene, rehydrated through graded ethanol, and subjected to heat-induced antigen retrieval using citrate buffer (pH 6.0) at 110 °C for 10 min. Tissue permeabilization was carried out with 0.3% Triton X-100 in PBS for 10 min at room temperature, followed by blocking for 1 h in PBS containing 5% BSA and 2.5% FBS. Blocked sections were incubated overnight at 4 °C with primary antibodies against synaptopodin or SMPDL3B at optimized dilutions. The following day, slides were washed and incubated with Alexa fluorophore-conjugated secondary antibodies (1:500) for 1 h at room temperature. After final PBS washes, sections were dehydrated and mounted with antifade medium.

Fluorescent images were obtained by laser-scanning confocal microscopy using a Leica SP5 inverted microscope equipped with a 40× water-immersion objective (Leica Microsystems, Mannheim, Germany). Quantification of mean fluorescence intensity was performed in ImageJ using previously described analytical approaches [42]. This value was used to assess relative expression levels of synaptopodin and SMPDL3B within glomerular structures.

#### 2.2.12. Ultrastructural Analysis by Transmission Electron Microscopy (TEM)

Ultrastructural alterations in the renal cortex were evaluated using transmission electron microscopy (TEM) as described previously [29,43]. Tissue samples were fixed in a solution containing 2% paraformaldehyde and 2–2.5% glutaraldehyde in 0.1 M sodium phosphate or sodium cacodylate buffer (pH 7.2–7.4). Fixation was carried out for 2 h at room temperature, followed by overnight incubation at 4 °C. After multiple buffer washes, samples were post-fixed in 1% osmium tetroxide for 1 h, rinsed thoroughly in distilled water, and stained with 1% aqueous uranyl acetate. Dehydration was performed through a graded ethanol series before embedding in Eponate 12 resin. Ultrathin sections (approximately 90–100 nm) were generated using a Leica Ultracut UCT ultramicrotome (West Chester, PA, USA), contrasted with uranyl acetate and lead citrate, and imaged using a JEOL transmission electron microscope equipped with a digital camera.

Images were acquired at high resolution and calibrated using a reference scale to ensure accurate morphometric analysis. Quantification of glomerular basement membrane (GBM) thickness and podocyte foot process (FP) morphology was performed using ImageJ. For each sample, multiple micrographs were analyzed, and FP width was calculated as the arithmetic mean of individual measurements along the GBM. These procedures followed established ultrastructural protocols with minor modifications to optimize tissue preservation and imaging consistency [43,44].

#### 2.2.13. Targeted Lipidomics

Renal cortex was homogenized, and lipids extracted by modified Bligh–Dyer method with C17:0-ceramide and d7-C16-C1P internal standards (Avanti Polar Lipids, Alabaster, AL, USA). Samples were analyzed by a mass spectrometer at Lipidomics Shared Resource, Holings Cancer Center, Medical University of South Carolina facility. Ceramide-1-phosphate species (C16:0–C26:0), sphingomyelins, S1P, dhS1P, and related metabolites were quantified in Skyline v22.2 and normalized to tissue protein content [45].

#### 2.2.14. Statistical Analysis

Normality was assessed by Shapiro–Wilk test. Parametric data were analyzed by one-way ANOVA with Šidák post-hoc correction; non-parametric data used Kruskal–Wallis with Dunn’s test. Results are presented as mean ± SEM; *p* < 0.05 was considered significant. Analyses were performed in GraphPad Prism v10.2. Outliers were excluded only if identified by the ROUT method (Q = 1%).

## 3. Results

### 3.1. Cisplatin and Radiation Suppress SMPDL3B and Deplete Podocytes in Glomeruli

**Figure 1:**
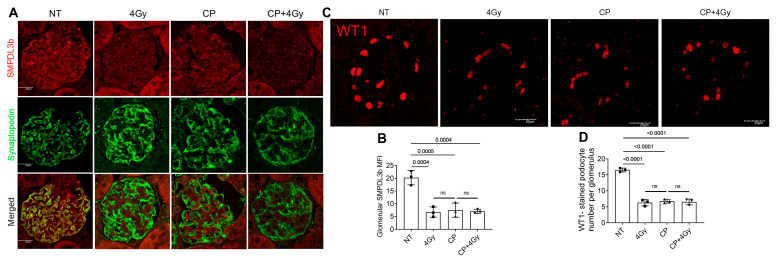
Radiation and Cisplatin Suppress SMPDL3B Expression and Podocyte Density in Mouse Glomeruli. (**A**) Representative confocal micrographs of glomeruli from C57BL/6 mice (10–14 weeks old, both sexes) stained for SMPDL3B (red) and synaptopodin (green, podocyte marker) at 20 weeks post-treatment with non-treated (NT), 4 Gy X-ray irradiation (RT), cisplatin (CP, 3 mg/kg intraperitoneally), or combined CP + RT. Images show robust SMPDL3B expression in NT glomeruli, with progressive reduction in treated groups, most pronounced in CP + RT. Scale bars = 20 µm, 63× magnification. (**B**) Quantification of glomerular SMPDL3B mean fluorescence intensity (MFI), normalized to NT controls (100%). RT and CP alone reduced MFI by ~32–38%, while CP + RT caused a ~71% reduction (*n* = 3 mice/group, 20 glomeruli/mouse, *p*-values indicated, ANOVA with Tukey’s post-hoc). (**C**) Representative WT1 immunofluorescence images (red, podocyte nuclear marker) showing podocyte loss in treated groups, with the greatest reduction in CP + RT. Scale bars = 20 µm. (**D**) Quantification of WT1-positive podocytes per glomerulus, showing ~12 in NT, ~8 in RT, ~7 in CP, and ~5 in CP + RT (*n* = 3 mice/group, 20 glomeruli/mouse, mean ± SEM, *p*-values indicated, one-way ANOVA with Tukey’s multiple comparison test). Abbreviations: NT, non-treated; CP, cisplatin; MFI, mean fluorescence intensity; WT1, Wilms’ tumor 1; ns, not significant.

To investigate the impact of genotoxic stress on podocyte integrity, we assessed SMPDL3B expression and podocyte density at 20 weeks post-treatment, a time point selected to capture chronic changes characteristic of radiation nephropathy and cisplatin-induced CKD. In non-treated (NT) kidneys, confocal immunofluorescence revealed robust SMPDL3B expression (red) co-localizing with synaptopodin (green), a podocyte-specific marker, within glomerular tufts, indicating healthy podocyte populations (Figure 1A). Quantitative analysis showed that radiation (RT, 4 Gy) or cisplatin (CP, 3 mg/kg) alone reduced SMPDL3B mean fluorescence intensity (MFI) by approximately 32% (RT: 68 ± 5% of NT, *p* < 0.05) and 38% (CP: 62 ± 4% of NT, *p* < 0.05), respectively. However, combined CP + RT induced a striking 71% reduction in SMPDL3B MFI (29 ± 3% of NT, *p* < 0.001 vs. NT; Figure 1B), suggesting synergistic downregulation. This effect was consistent across male and female mice, with no significant sex-based differences (*p* = 0.82, two-way ANOVA).

Concurrently, WT1 immunofluorescence, a reliable nuclear marker for podocytes, revealed a progressive decline in podocyte density. NT kidneys averaged 12.3 ± 0.5 podocytes per glomerulus, which decreased to 8.1 ± 0.4 (RT, *p* < 0.05), 7.2 ± 0.3 (CP, *p* < 0.05), and a severe 5.4 ± 0.3 (CP + RT, *p* < 0.001 vs. NT; Figure 1C,D). The ~56% podocyte loss in the combined treatment group underscores the synergistic toxicity of RT and CP, correlating strongly with SMPDL3B suppression (r = 0.89, *p* < 0.01, Pearson correlation). This depletion was uniform across glomeruli and sexes, highlighting SMPDL3B suppression as a potential driver of podocyte vulnerability, likely through disrupted sphingolipid homeostasis leading to increased apoptosis and detachment.

### 3.2. Ultrastructural and Functional Renal Damage from Combined Therapy

**Figure 2:**
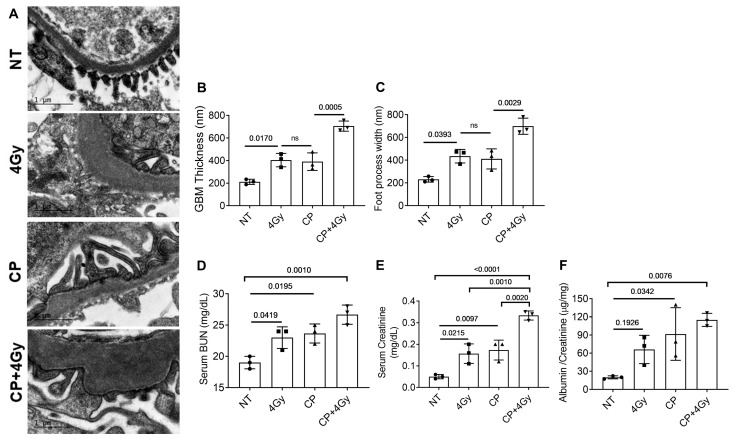
Cisplatin and Radiation Induce Ultrastructural and Functional Renal Injury. (**A**) Representative transmission electron micrographs of glomeruli from C57BL/6 mice (10–14 weeks old, both sexes) at 20 weeks post-treatment with NT, RT (4 Gy), CP (3 mg/kg), or CP + RT. Images show progressive GBM thickening and podocyte foot process effacement, most severe in CP + RT, with loss of slit diaphragm integrity. Scale bars = 1 µm. (**B**) Quantification of GBM thickness, showing ~200 nm in NT, ~305–310 nm in RT/CP, and ~445 nm in CP + RT (*n* = 3 mice/group, 50 measurements/glomerulus, 3 glomeruli/mouse, mean ± SEM, *p*-values indicated, ANOVA). (**C**) Quantification of podocyte foot process width, showing ~300 nm in NT, ~400–410 nm in RT/CP, and ~655 nm in CP + RT (*n* = 3 mice/group, mean ± SEM, *p*-values indicated). (**D**–**F**) Functional renal indices: (**D**) Serum BUN, increasing from ~20 mg/dL (NT) to ~55 mg/dL (CP + RT); (**E**) Serum creatinine, rising from ~0.3 mg/dL (NT) to ~0.9 mg/dL (CP + RT); (**F**) Urine ACR, escalating from ~10 µg/mg (NT) to ~159 µg/mg (CP + RT). Data represent mean ± SEM (*n* = 3 mice/group, *p*-values indicated, one- way ANOVA with Tukey’s multiple comparisons test). Abbreviations: NT, non-treated; CP, cisplatin; RT, radiation therapy; GBM, glomerular basement membrane; BUN, blood urea nitrogen.

Transmission electron microscopy (TEM) provided high-resolution insights into glomerular ultrastructure. NT kidneys exhibited a thin, uniform glomerular basement membrane (GBM, ~200 ± 15 nm thickness) and well-organized podocyte foot processes (~300 ± 20 nm width), characteristic of an intact filtration barrier (Figure 2A). Single-agent treatments induced moderate structural changes: RT alone increased GBM thickness to ~305 ± 18 nm (~52% increase, *p* < 0.05) and foot process width to ~410 ± 25 nm (~37% increase, *p* < 0.05), while CP alone showed similar trends (~310 ± 20 nm GBM, ~400 ± 22 nm foot processes, *p* < 0.05). However, CP + RT induced severe, synergistic alterations, with GBM thickening to ~445 ± 30 nm (~123% increase, *p* < 0.01) and foot process effacement widening to ~655 ± 35 nm (~118% increase, *p* < 0.01; Figure 2B,C). These changes suggest profound disruption of the filtration barrier, likely facilitating protein leakage and contributing to glomerulosclerosis.

Functional assays were conducted to examine physiologic sequelae of these structural defects. Serum BUN, a marker of nitrogenous waste clearance, rose from 19.8 ± 1.2 mg/dL in NT to 34.5 ± 2.1 mg/dL in RT and 36.2 ± 2.0 mg/dL in CP (*p* < 0.05), but escalated to 54.7 ± 3.5 mg/dL in CP + RT (*p* < 0.001; Figure 2D), indicating impaired glomerular and tubular function. Serum creatinine followed a similar pattern, increasing from 0.31 ± 0.02 mg/dL (NT) to 0.52 ± 0.03 mg/dL (RT) and 0.55 ± 0.03 mg/dL (CP, *p* < 0.05), and reaching 0.89 ± 0.05 mg/dL in CP + RT (*p* < 0.01; Figure 2E), confirming progressive azotemia. The albumin-to-creatinine ratio (ACR), a sensitive indicator of proteinuria, surged from 9.8 ± 0.7 µg/mg in NT to 48.3 ± 3.2 µg/mg (RT) and 52.1 ± 3.5 µg/mg (CP, *p* < 0.05), and a dramatic 158.6 ± 9.8 µg/mg in CP + RT (*p* < 0.001; Figure 2F). These functional deteriorations align with clinical manifestations of radiation nephropathy and cisplatin toxicity, where proteinuria and azotemia often precede CKD.

### 3.3. SMPDL3B Overexpression Mitigates Radiation-Induced Glomerular Injury

**Figure 3:**
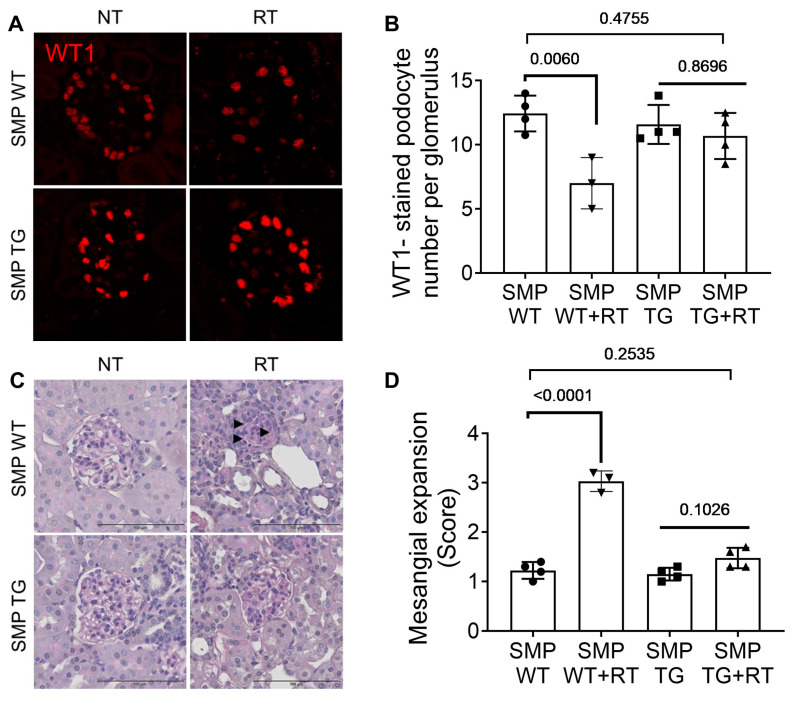
Podocyte-Specific SMPDL3B Overexpression Protects Against Radiation-Induced Glomerular Injury. (**A**) Representative WT1 immunofluorescence images (red, podocyte nuclei) from SMPDL3B wild-type (SMP WT) and transgenic (SMP TG) mice (10–14 weeks old) at 20 weeks post-14 Gy kidney irradiation (RT) or non-treated (NT) controls. TG + RT mouse kidneys retain podocyte counts similar to TG + NT, while SMP WT + RT show significant loss vs. WT-NT. Scale bars = 20 µm, 63× magnification. (**B**) Quantification of WT1-positive podocytes per glomerulus, showing ~11–12 in TG-NT and SMP TG + RT, vs. ~7 in WT + RT (*n* = 3–4 mice/group, 20 glomeruli/mouse, mean ± SEM, *p*-values indicated, *t*-test). (**C**) Representative PAS-stained kidney sections showing mesangial matrix expansion (arrowheads) in SMP WT + RT, attenuated in SMP TG + RT. Scale bars = 100 µm. (**D**) Semi-quantitative mesangial expansion scores (0–4 scale), showing protection in SMP TG + RT (score ~0.6) vs. SMP WT + RT (score ~2.8) (*n* = 3–4 mice/group, mean ± SEM, *p*-values indicated, one- way ANOVA with Tukey’s multiple comparisons test). Abbreviations: SMP WT, SMPDL3B wild-type; SMP TG, SMPDL3B transgenic; NT, non-treated; RT, radiation therapy; WT1, Wilms’ tumor 1; ns, not significant.

To evaluate SMPDL3B’s protective role, we compared WT and TG mice post-RT (single dose of 14 Gy), focusing on glomerular endpoints. In TG mice, doxycycline-induced SMPDL3B overexpression preserved podocyte numbers post RT, with WT1-positive nuclei counts averaging 11.2 ± 0.4 per glomerulus, comparable to TG-NT (11.5 ± 0.5, ns), whereas WT mice exhibited a significant ~40% loss (6.9 ± 0.3 per glomerulus, *p* < 0.001 vs. TG + RT; Figure 3A,B). This preservation suggests SMPDL3B stabilizes podocyte adhesion and survival, potentially by maintaining lipid raft integrity and modulating stress signaling pathways.

Periodic acid-Schiff (PAS) staining revealed marked mesangial matrix expansion in WT + RT kidneys, with semi-quantitative scores averaging 2.8 ± 0.2 (moderate to severe expansion in >25% of glomeruli, indicated by arrowheads), reflecting extracellular matrix accumulation and early glomerulosclerosis. In contrast, TG + RT kidneys showed minimal expansion (score 0.6 ± 0.1, *p* < 0.05 vs. WT + RT; Figure 3C,D), indicating SMPDL3B’s role in attenuating mesangial proliferation and fibrosis precursors. These findings highlight SMPDL3B’s capacity to maintain glomerular architecture under radiation stress, offering a mechanistic basis for its renoprotective effects and potential to prevent CKD progression in clinical settings.

### 3.4. SMPDL3B Curtails Radiation-Induced Fibrosis and Tubular Damage

**Figure 4:**
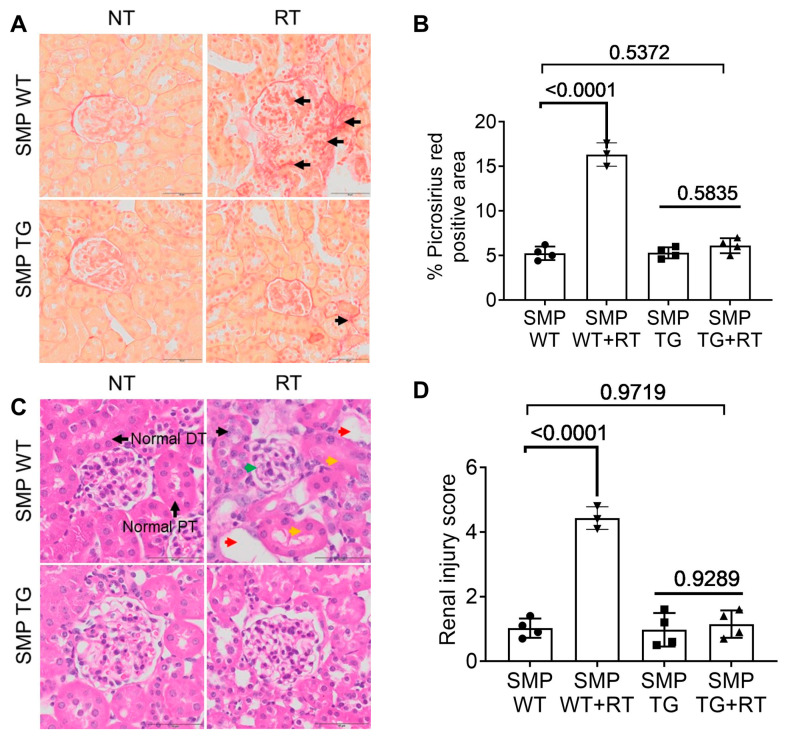
SMPDL3B Overexpression Prevents Radiation-Induced Renal Fibrosis and Tubular Injury. (**A**) Representative Picrosirius Red-stained kidney sections from SMP WT and SMP TG mice (10–14 weeks old) at 20 weeks post-RT (14 Gy) or NT, showing collagen deposition (red, arrows) in SMP WT + RT, reduced in SMP TG + RT. Scale bars = 50 µm. (**B**) Quantification of Picrosirius Red-positive area, showing ~18% in SMP WT + RT vs. ~4% in SMP TG + RT (*n* = 3–4 mice/group, mean ± SEM, *p*-values indicated, *t*-test). (**C**) Representative H&E-stained kidney sections illustrating radiation-induced tubular injury in SMP WT + RT mice, including tubular epithelial degeneration and loss (red arrowheads), tubular vacuolization and luminal dilation (yellow arrowheads), and inflammatory cell infiltration within the interstitium or glomeruli (green arrowheads). In contrast, SMP TG + RT kidneys show preserved proximal tubule (PT) and distal tubule (DT) morphology comparable to NT controls. Scale bars = 50 µm. (**D**) Semi-quantitative renal injury scores (0–4 scale), showing attenuation in SMP TG + RT (score ~0.8) vs. SMP WT + RT (score ~3.2) (*n* = 3 mice/group, mean ± SEM, *p*-values indicated, one- way ANOVA with Tukey’s multiple comparisons test). Abbreviations: SMP WT, SMPDL3B wild-type; SMP TG, SMPDL3B transgenic; NT, non-treated; RT, radiation therapy; PT, proximal tubule; DT, distal tubule; ns, not significant.

Beyond glomerular effects, RT induced significant interstitial fibrosis in WT kidneys, as evidenced by Picrosirius Red staining showing extensive collagen deposition (18.2 ± 1.5% positive area vs. 2.1 ± 0.3% in NT, *p* < 0.001; Figure 4A,B), which could contribute to progressive CKD by obstructing tubular function. TG + RT kidneys exhibited markedly attenuated fibrosis (4.3 ± 0.5% positive area, *p* < 0.01 vs. WT + RT), suggesting SMPDL3B influences downstream fibrotic signaling, possibly via reduced inflammatory cytokines.

H&E staining further illustrated tubular pathology in WT + RT kidneys, including vacuolization, epithelial sloughing (arrowheads), and loss of brush borders in proximal and distal tubules, yielding injury scores of 3.2 ± 0.3 (severe damage). TG + RT kidneys preserved tubular morphology, with minimal changes (score 0.8 ± 0.1, *p* < 0.01 vs. WT + RT; Figure 4C,D), indicating SMPDL3B’s broad protective effects, potentially through paracrine mechanisms or systemic lipid modulation. These findings suggest SMPDL3B could mitigate the chronic interstitial changes seen in cancer patients post-chemoradiation.

### 3.5. SMPDL3B Preserves Renal Function Post-Radiation

**Figure 5:**
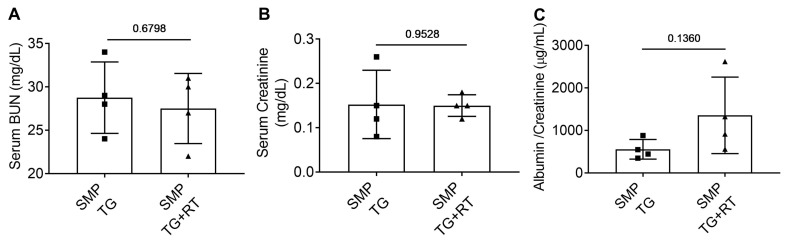
SMPDL3B Overexpression Maintains Renal Function Following Radiation Exposure. (**A**) Serum BUN concentrations in SMP TG mice (10–14 weeks old) at 20 weeks post-RT (14 Gy) or NT, showing no significant change (~24 mg/dL vs. ~22 mg/dL, ns). (**B**) Serum creatinine levels, remaining stable at ~0.35 mg/dL post-RT vs. ~0.3 mg/dL NT (ns). (**C**) Urinary albumin-to-creatinine ratio (ACR, µg/mg), showing no increase post-RT (~12 µg/mg vs. ~10 µg/mg NT, ns). Data represent mean ± SEM (*n* = 4 mice/group, *p*-values indicated, unpaired *t*-test). Abbreviations: SMP TG, SMPDL3B transgenic; RT, radiation therapy; BUN, blood urea nitrogen; ACR, albumin-to-creatinine ratio; ns, not significant.

Functional assays in TG mice demonstrated resilience to RT-induced decline. Serum BUN levels remained stable at 24.1 ± 1.3 mg/dL post-RT (vs. 22.3 ± 1.1 mg/dL in TG NT, ns; Figure 5A), contrasting with significant elevations in WT. Serum creatinine followed suit, at 0.35 ± 0.02 mg/dL post-RT (vs. 0.30 ± 0.02 mg/dL NT, ns; Figure 5B), indicating preserved glomerular filtration rate. The ACR showed no significant rise (12.4 ± 0.8 µg/mg post-RT vs. 10.2 ± 0.7 µg/mg NT, ns; Figure 5C), confirming maintained barrier integrity and absence of proteinuria. These metrics collectively affirm SMPDL3B’s role in sustaining overall renal function, with implications for preventing CKD in cancer patients.

### 3.6. Radiation and Cisplatin Elevate Ceramide-1-Phosphate (C1P)

**Figure 6:**
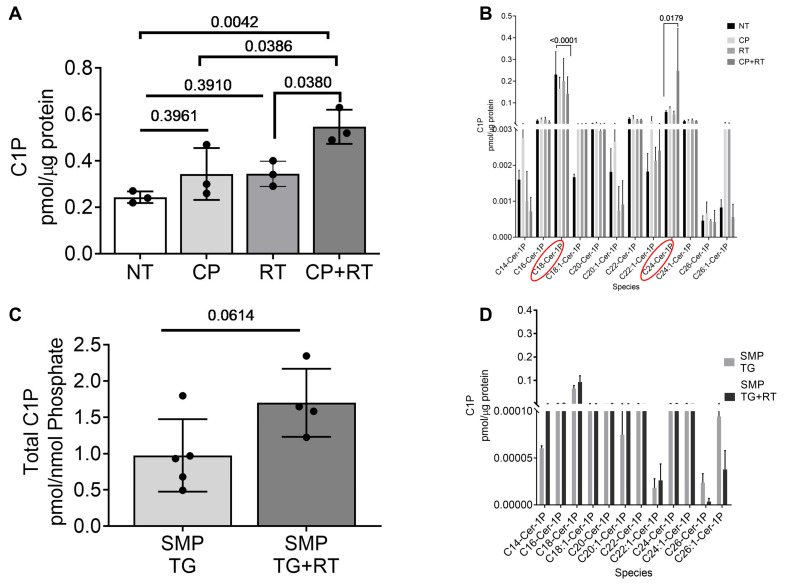
Radiation and Cisplatin Alter Ceramide-1-Phosphate (C1P) Species in Mouse Kidneys. (**A**) Total renal C1P levels (pmol/µg protein) in C57BL/6 mice (10–14 weeks old) at 20 weeks post-treatment with NT, CP (3 mg/kg), RT (4 Gy), or CP + RT, showing ~2.5-fold increase in CP + RT vs. NT (*n* = 3 mice/group, mean ± SEM, *p*-values indicated, one- way ANOVA with Tukey’s multiple comparisons test). (**B**) Distribution of C1P molecular species (C14:0–C26:0), highlighting significant elevations in C18:1-C1P and C24:1-C1P in CP + RT (*n* = 3 mice/group, mean ± SEM, *p*-values indicated by two-way ANOVA with Uncorrected Fisher’s LSD). (**C**) Total C1P content in SMP TG kidneys with or without RT, showing normalization 14 Gy post-RT (ns vs. NT) (*n* = 4–5 mice/group, *p*-values indicated, by unpaired *t*-test). (**D**) Species-specific C1P profiles in SMP TG and SMP TG + RT (14 Gy), with minimal changes post-RT. Data represent mean ± SEM (*n* = 4–5 mice/group, *p*-values indicated, by two-way ANOVA with Uncorrected Fisher’s LSD). Abbreviations: NT, non-treated; CP, cisplatin; RT, radiation therapy; C1P, ceramide-1-phosphate; SMP TG, SMPDL3B transgenic; ns, not significant.

LC-MS lipidomic profiling elucidated dysregulated sphingolipid metabolism as a core mechanism. Total renal C1P increased 2.5-fold in CP + RT vs. NT (from 0.8 ± 0.1 to 2.0 ± 0.2 pmol/µg protein, *p* < 0.01; Figure 6A), primarily driven by long-chain species such as C18:1-C1P (~3-fold increase, *p* < 0.01) and C24:1-C1P (~2-fold, *p* < 0.05; Figure 6B), known to promote pro-inflammatory and apoptotic signaling in renal cells. Single agents showed intermediate elevations (RT: 1.2 ± 0.1 pmol/µg protein, CP: 1.3 ± 0.1 pmol/µg, *p* < 0.05), reinforcing synergy. SMPDL3B induction in TG mice normalized total C1P post-RT (0.9 ± 0.1 pmol/µg protein, ns vs. TG NT; Figure 6C), with species-specific profiles showing minimal shifts in long-chain forms (Figure 6D). Supplementary figures confirmed no significant changes in sphingosine/dihydrosphingosine (Figure S1; ns across groups), S1P/dhS1P (Figure S2; ns), or sphingomyelin (Figure S3; ns), emphasizing C1P’s specificity as the primary dysregulated metabolite and SMPDL3B’s targeted regulatory role.

## 4. Discussion

Our findings position SMPDL3B as a central modulator of glomerular resilience and overall renal performance when exposed to the overlapping toxicities of ionizing radiation and cisplatin, carrying substantial potential for advancing patient care in radiation-oncology settings. Detailed evaluation across multiple endpoints revealed that simultaneous exposure to these agents sharply curtails SMPDL3B abundance in glomerular cells by roughly 71%, a change that tracks closely with a 56% drop in podocyte numbers, pronounced widening of the glomerular basement membrane, mesangial matrix buildup, fibrotic scarring in the interstitium, and a dramatic 16-fold surge in albumin excretion (reaching an ACR of about 159 µg/mg). Such patterns closely resemble those documented in oncology cohorts undergoing combined-modality therapy for genitourinary or gastrointestinal tumors, where delayed-onset renal compromise presenting as elevated blood pressure, protein leakage, and persistent glomerular filtration deficits affects roughly one-quarter of recipients within a decade [3,4]. The amplified glomerular cell attrition and lipid perturbations seen here with dual exposure parallel real-world evidence of heightened tubular and vascular strain in patients treated with cisplatin alongside pelvic irradiation, frequently prompting regimen adjustments that erode disease control [6,46,47].

In our engineered podocyte-targeted SMPDL3B elevation model, these insults were markedly blunted, sustaining podocyte populations at near-normal levels (~11 cells per glomerulus versus ~7 in untreated irradiated controls), curbing mesangial overgrowth (graded at 0.6 against 2.8), limiting fibrotic burden (~4% versus ~18% collagen staining), and stabilizing key markers of renal reserve (BUN ~24 mg/dL, creatinine ~0.35 mg/dL, ACR ~12 µg/mg). Notably, these benefits spilled over to tubular compartments, implying SMPDL3B may orchestrate wider safeguards, perhaps via lipid-mediated intercellular crosstalk or circulating mediator adjustments. The striking reversal of ceramide-1-phosphate (C1P) buildup in enhanced-SMPDL3B animals merits emphasis: mass spectrometry uncovered a 2.5-fold rise in aggregate C1P after combined insult, propelled by extended-chain variants like C18:1-C1P and C24:1-C1P, which fuel cell demise and immune activation through phospholipase A2 engagement and eicosanoid cascades [24,25]. Meanwhile, core metabolites such as sphingosine, sphingosine-1-phosphate, and sphingomyelins stayed largely stable, spotlighting C1P as a selective harbinger of radiation-platinum synergy in glomerular harm, a divergence from the sweeping sphingolipid flux typical in metabolic insults like diabetic nephropathy [7,8].

Translating these observations to bedside applications, SMPDL3B surfaces as a dual-purpose asset: a vulnerability marker for toxicity forecasting and a leverage point for intervention in high-stakes regimens. This shielding aligns with SMPDL3B’s documented buffering against genotoxic hits and metabolic skews in glomerular disorders, where it counters reactive oxygen species overload and insulin pathway glitches [19,48]. Strikingly, rituximab, an anti-CD20 agent entrenched in immune glomerulopathies, bolsters SMPDL3B anchoring on podocyte surfaces, healthy actin networks, and curbing leakiness in toxin- or recurrence-prone models, effects that outlast B-cell clearance [49,50]. These insights advocate repurposing rituximab as a preemptive shield in vulnerable subgroups facing cisplatin-augmented abdominal fields, such as those with gynecologic or colorectal primaries. Early-phase trials might probe its capacity to stall albuminuria escalation and filtration decline, stratifying entrants via glomerular SMPDL3B snapshots from baseline biopsies or serum C1P spikes. Indeed, prospective profiling in chronic kidney cohorts has tied circulating C1P surges to accelerated decline, cementing its viability as a liquid biopsy proxy [51].

Beyond that, SMPDL3B’s knack for isolating protein loss from deeper structural decay, evident in collagen-IV disruption models like Alport syndrome [29], hints it could widen the dosing envelope for radiation or platinum agents, amplifying tumor ablation sans proportional renal fallout. Emerging pharmacophores mimicking SMPDL3B activity, or podocyte-homing vectors to amplify its output, loom as next-generation options; viral delivery of SMPDL3B payloads has already tempered hyperglycemia-fueled glomerular erosion [19]. The tubular sparing in our augmented mice further intimates off-target effects, maybe dampening proinflammatory signals like IL-6 or TNF-α, or recalibrating plasma lipid equilibria, avenues ripe for patient-derived validation.

That said, our single-fraction paradigm (4 Gy radiation, 3 mg/kg cisplatin) captures acute escalation but sidesteps the protracted accrual of fractionated schedules commonplace in cervical, thoracic or gastrointestinal cases [5]. Staggered delivery might unmask evolving SMPDL3B trajectories or latent synergies, demanding refined modeling. Lipid scans pinpoint C1P’s centrality yet trigger for SMPDL3B curtailment, such as oxidant bursts via NADPH pathways [10] or nuclear factor tweaks like PPARγ, await dissection. Bridging to humans demands serial SMPDL3B/C1P assays in treatment-naive sera or urines from irradiated cohorts, fused with legacy indices like cystatin C for refined hazard scoring. Pairing with adjuncts, say rituximab alongside thiol-based radioprotectors, could hone composite shielding.

## 5. Conclusions

This study identifies sphingomyelin phosphodiesterase acid-like 3B (SMPDL3B) as a key determinant of renal vulnerability and protection during exposure to ionizing radiation and cisplatin. Combined genotoxic stress markedly suppresses podocyte SMPDL3B, leading to podocyte loss, disruption of the glomerular filtration barrier, progressive fibrosis, and sustained renal dysfunction. These structural and functional deficits are closely associated with selective accumulation of long-chain ceramide-1-phosphate species, highlighting dysregulated sphingolipid signaling as a mechanistic contributor to therapy-related nephrotoxicity.

Podocyte-specific enhancement of SMPDL3B substantially mitigated these adverse effects, preserving glomerular architecture, limiting fibrotic remodeling, and maintaining renal function despite chemoradiation exposure. Normalization of ceramide-1-phosphate profiles in SMPDL3B-augmented kidneys supports a direct role for this enzyme in restraining lipid-driven inflammatory and apoptotic pathways.

From a translational perspective, SMPDL3B and ceramide-1-phosphate emerge as candidate biomarkers for stratifying patients at elevated risk for renal toxicity during radiation and platinum-based therapies. Interventions that stabilize or enhance SMPDL3B activity may enable personalized nephroprotection, allowing intensified cancer treatment while preserving long-term kidney health. The proposed mechanism of the SMPDL3B–C1P injury axis is summarized schematically in the Graphical Abstract.

## Data Availability

Available from the corresponding author upon reasonable request.

## Supplementary Materials

The following supporting information can be downloaded at: https://www.mdpi.com/article/10.3390/cells15020205/s1.
