# Supplementary material for: Targeting SMPDL3B to Ameliorate Radiation- and Cisplatin-Induced Renal Toxicity
Source: Cells. 2026 Jan 22;15(2):205. doi: 10.3390/cells15020205 (PMC12839682; doi:10.3390/cells15020205)
Supplement: Supplementary file 1 [file cells-15-00205-s001.zip › cells-4095945-supplementary.pdf]

## Figure legends

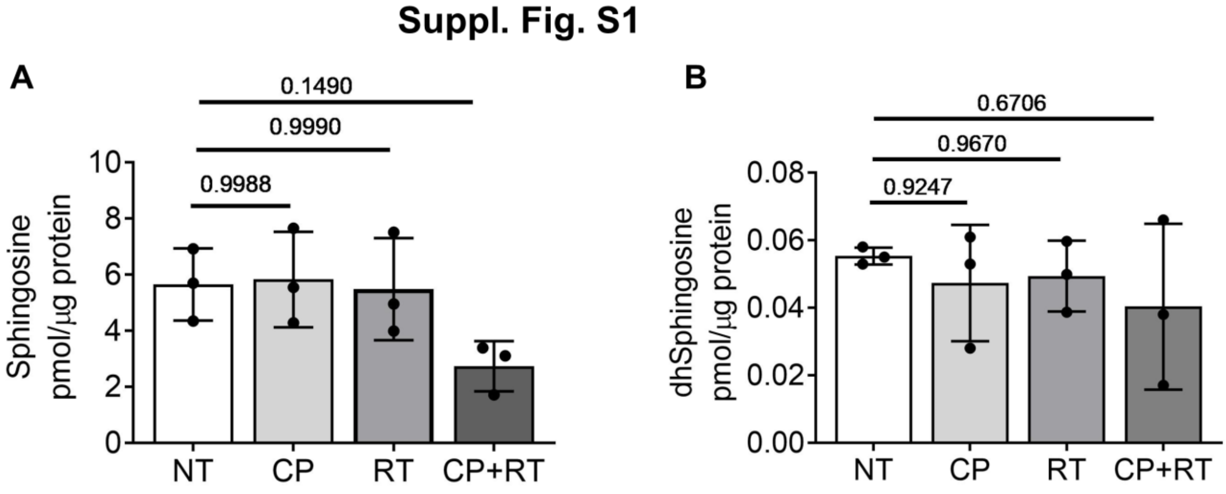


**Supplementary Figure S1. Sphingosine and Dihydrosphingosine Levels Remain Unchanged.** (A) Sphingosine levels (pmol/µg protein) in C57BL/6 mice (10–14 weeks old) at 20 weeks post-treatment with NT, CP, RT, or CP + RT, showing no significant changes (n=3 mice/group, mean ± SEM, ns, ANOVA). (B) Dihydrosphingosine (dhSphingosine) concentrations, also unchanged across groups (n=3 mice/group, mean ± SEM, ns, one- way ANOVA with Tukey's multiple comparisons test). **Abbreviations:** NT, non-treated; CP, cisplatin; RT, radiation therapy; LC-MS, liquid chromatography–mass spectrometry.

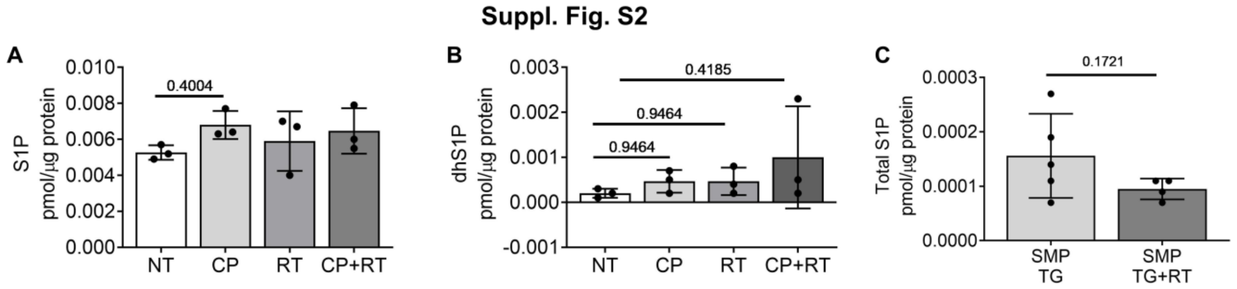


**Supplementary Figure S2: Minimal Effects on Sphingosine-1-Phosphate (S1P) and Dihydrosphingosine-1-Phosphate (dhS1P).** (A) Total S1P content (pmol/µg protein) in C57BL/6 mice (10–14 weeks old) at 20 weeks post-treatment with NT, CP, RT, or CP + RT, showing no significant differences (n=3–5 mice/group, mean ± SEM, ns, ANOVA). (B) dhS1P concentrations across the same groups, also unchanged (n=3–5 mice/group, ns). (C) Total S1P levels in SMP TG kidneys with or without RT, showing no differences (n=3-5mice/group, ns, t-test). **Abbreviations:** NT, non-treated; CP, cisplatin; RT, radiation therapy; S1P, sphingosine-1-phosphate; dhS1P, dihydrosphingosine-1-phosphate; SMP TG, SMPDL3B transgenic; ns, not significant.

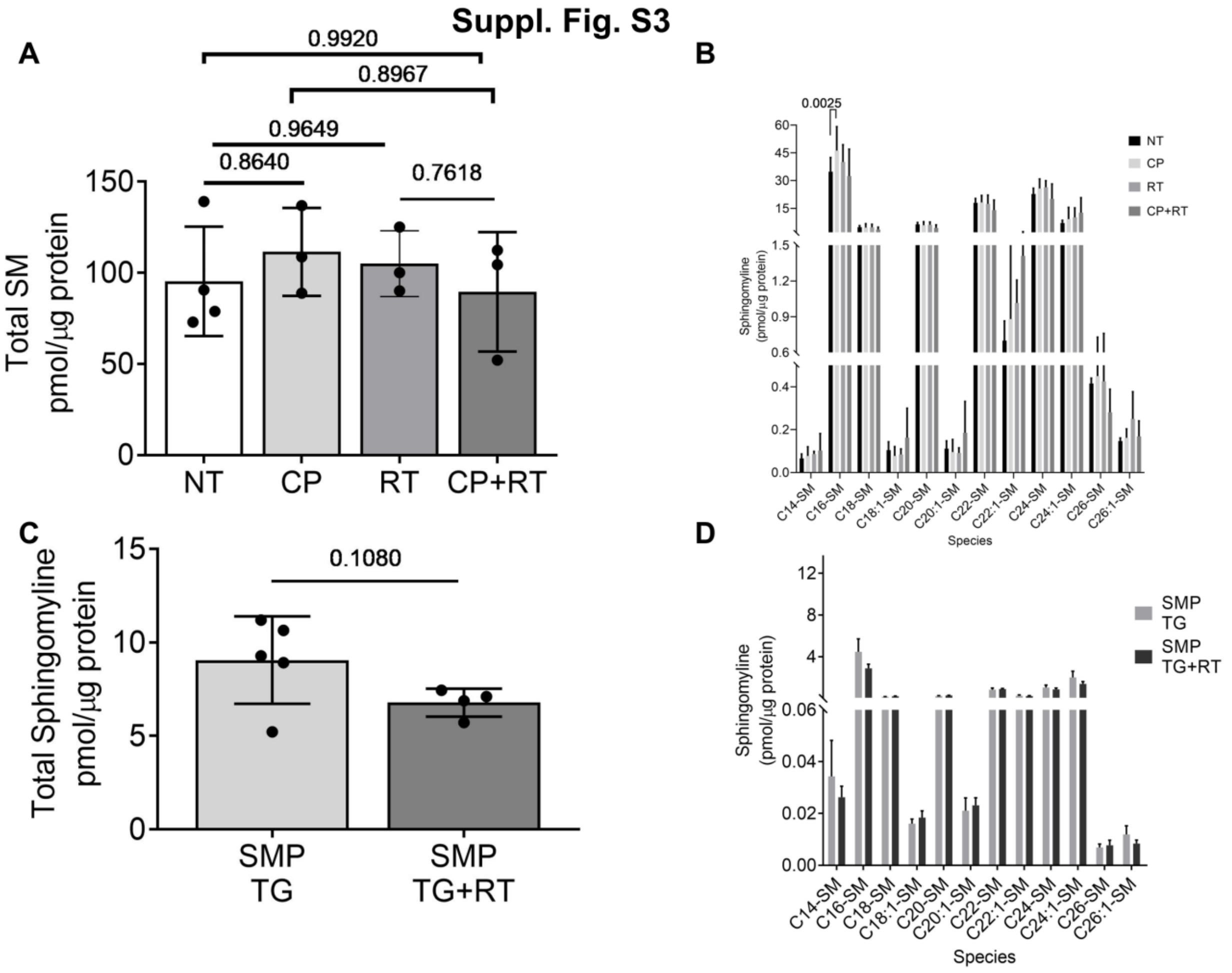


**Supplementary Figure S3: No Significant Changes in Sphingomyelin Levels.** (A) Total sphingomyelin (SM) content (pmol/µg protein) in C57BL/6 mice (10–14 weeks old) at 20 weeks post-treatment with NT, CP, RT, or CP + RT, showing no consistent changes (n=3–4 mice/group, mean ± SEM, ns, ANOVA). (B) Distribution of SM molecular species (C14:0–C26:0), with minor variations in C16:1-SM but no treatment-dependent pattern. (C) Total SM content in SMP TG kidneys with or without RT, showing no differences. (D) Species-specific SM profiles in SMP TG and SMP TG + RT, with no significant changes. Data represent mean ± SEM (n=3–5 mice/group, P-values indicated, t-test). **Abbreviations:** NT, non-treated; CP, cisplatin; RT, radiation therapy; SM, sphingomyelin; SMP TG, SMPDL3B transgenic; ns, not significant.
